# Psychosocial Functioning in Depressive Patients: A Comparative Study between Major Depressive Disorder and Bipolar Affective Disorder

**DOI:** 10.1155/2014/302741

**Published:** 2014-03-13

**Authors:** Shubham Mehta, Pankaj Kumar Mittal, Mukesh Kumar Swami

**Affiliations:** ^1^Department of Psychiatry, SMS Medical College, Jaipur, Rajasthan 302004, India; ^2^Department of Psychiatry, All India Institute of Medical Sciences, Bhopal, Madhya Pradesh 462024, India; ^3^Department of Psychiatry, BPS Government Medical College for Women, Sonepat, Haryana 131305, India

## Abstract

*Introduction*. Major depressive disorder (MDD) and bipolar affective disorder (BAD) are among the leading causes of disability. These are often associated with widespread impairments in all domains of functioning including relational, occupational, and social. The main aim of the study was to examine and compare nature and extent of psychosocial impairment of patients with MDD and BAD during depressive phase. *Methodology*. 96 patients (48 in MDD group and 48 in BAD group) were included in the study. Patients were recruited in depressive phase (moderate to severe depression). Patients having age outside 18–45 years, psychotic symptoms, mental retardation, and current comorbid medical or axis-1 psychiatric disorder were excluded. Psychosocial functioning was assessed using Range of Impaired Functioning Tool (LIFE-RIFT). *Results*. Domains of work, interpersonal relationship, life satisfaction, and recreation were all affected in both groups, but the groups showed significant difference in global psychosocial functioning score only (*P* = 0.031) with BAD group showing more severe impairment. *Conclusion*. Bipolar depression causes higher global psychosocial impairment than unipolar depression.

## 1. Introduction

According to World Health Organization (WHO), depression is the leading cause of disability as measured by Years Lived with Disability (YLDs) and disability due to depression exceeds disability due to all forms of cancer and diabetes mellitus combined, as well as the disability due to stroke and hypertensive heart diseases [1]. Global Burden of Disease 2000 study showed that depression accounts for 4.46% of total Disability Adjusted Life Years (DALYs) and 12.1% of total YLDs [2]. This clearly highlights paramount burden of disability which occurs secondary to depression. Much of this burden relates to the economic losses suffered (both personal and to society) when people are depressed and by impairment in their quality of life and relationships.

The physical, cognitive, and emotional symptom dimensions of depression lead to considerable impairment in psychosocial functioning. Psychosocial functioning reflects a person's ability to perform the activities of daily living and to engage in relationships with other people in ways that are gratifying to him and others, and that meets the demands of the community in which the individual lives. The relationship between physical depressive symptoms and impaired physical activity can be attributed to the fact that depressive episodes are defined by three symptoms relevant to physical activity including, decreased interest or pleasure in almost all activities throughout the day, psychomotor agitation or retardation, and fatigue or loss of energy nearly every day.

The cognitive symptoms of depression interfere with work. Three ways in which depression may impair work performance are (1) interpersonal relationships (depressed people are seen as irritable, pessimistic, and withdrawn); (2) productivity (less productive due to fatigue, poor decision-making, and lack of concentration); and (3) safety (greater risk of accidents or injuries among depressed people) [3].

Depressed people are often dependent on others emotionally and seek reassurance in ways that distance others. They may often overvalue relationships as sources of self-worth but may also acquire negative beliefs about the availability and faithfulness of others [4]. The interpersonal difficulties thereby may be persistent and recover more slowly than symptom changes [5].

Thus, it appears prudent to assess the functional impairment in depression as it is one of the leading causes of morbidity worldwide.

Like many other illnesses, depression also comes in different types and forms. However there are variations in severity, pattern of episodes, features, persistence of symptoms, and polarity characterizing different types of depression. There has been a continuous debate in literature regarding the subtyping of depression on basis of polarity into unipolar and bipolar depression. The issues between unipolar and bipolar depression, of similarities and differences in terms of etiology, symptoms, course, and outcomes, have guided the research for past several years.

As of now, only a few studies have evaluated the nature and extent of psychosocial deficits observed in depressed patients with unipolar and bipolar depression, especially in Indian context. We therefore carried out the present study with the aim to characterize and compare the psychosocial profiles of patients with major depressive disorder (MDD) and bipolar affective disorder (BAD) during depressive episodes. The comparison between the two disorders seemed to be rational, also because it has been shown that depression is the predominant mood symptom in bipolar disorders [6].

## 2. Methodology

The sample for this cross-sectional study was drawn from the patient attending outpatient department of psychiatric centre, Jaipur. We recruited 96 patients (aged from 18 to 45 years) in total, 48 patients meeting the criteria for an ICD-10 [7] diagnosis of MDD (unipolar) in one group and 48 patients meeting the ICD-10 diagnostic criteria for BAD (depressed) in second group. All patients were recruited during the depressive episode which had to be nonpsychotic and moderate to severe in nature as assessed by Hamilton Depression Rating Scale-17 item (HDRS-17 score ≥ 14) [8]. Exclusion criteria were current comorbid axis-I psychiatric disorder, neurological disorder, mental retardation, history of learning disability, and current/past history of drug abuse or dependence (except nicotine).

Study was approved by research review board and ethical committee of the institution. An informed consent was obtained from the subject prior to participation in the study.

After that, patients were screened on the basis of the inclusion and exclusion criteria. Those patients who satisfied the screening processes were recruited in the study.

Sociodemographic data (name, age, sex, marital status, education, occupation, monthly income, religion, type of family, and locality) and clinical profile including age of illness onset, total duration of illness, total number of affective episodes, number of depressive episodes, duration of current depressive episode, and family history of any psychiatric disorder were recorded.

Range of Impaired Functioning Tool (LIFE-RIFT) [9] was used to assess psychosocial functioning of the patients. It is a reliable and valid semistructured clinician-rated measure to psychosocial functioning in affective disorders. The RIFT consists of items from the Longitudinal Interval Follow-up Evaluation (LIFE). It has a total score and individual domain scores for the following four areas of functioning: work (employment, household, and school), interpersonal relations (spouse, children, other relatives, and friends), satisfaction, and recreation. Each domain of functioning is rated on a 5-point scale that includes behavioral anchors for each point: 1 = no impairment, very good functioning; 2 = no impairment, good functioning; 3 = mild impairment, fair functioning; 4 = moderate impairment, poor functioning; 5 = severe impairment, very poor functioning. For the work and interpersonal relation subscales, the ratings for the worst work and relationship categories were employed in LIFE-RIFT calculation. The score for the four domains were summed to generate a global score, ranging from 4 (no impairment, very good functioning) to 20 (severe impairment, very poor functioning).

*Statistical Analysis*. Statistical analysis was done with the help of Statistical Package for Social Sciences software (SPSS) version 20.0. Group comparison for sociodemographic variables and clinical variables was done with the help of application of chi-square test/Fisher's exact test and independent sample *t*-test, where appropriate. The psychosocial functioning variables were compared between groups using Mann-Whitney *U* test. The proportion of patients with significant psychosocial impairment was calculated. Patients were considered to have impairment in particular domain if their scores were at or above 3 by each domain and at or above 13 on total [10]. Out of the number of patients showing impairment in each psychosocial domain, percentage of patients showing mild, moderate, or severe impairment were, respectively, calculated.

## 3. Results

Mean age of MDD and BAD patients were 28.1 and 27.6, respectively. On application of independent sample *t*-test, no significant difference was found between groups (*P* = 0.649) for age (Table 1).

Most of the patients were males, married, educated from middle to senior secondary school, Hindu by religion, and coming from a nuclear family. Majority of patients were skilled/semiskilled/unskilled workers, earning between Rs. 6000 and 15000 per month. Both the groups were comparable with respect to all the sociodemographic variables (Table 1).

Mean duration of illness in MDD and BAD group was 15.3 months and 36.5 months, respectively, and the difference in both groups was statistically significant (*P* = 0.000). The BAD group had longer duration of illness because it included all the affective episodes (manic or depressive) and total number of affective episodes (manic and depressive) did not show significant correlation with global score of LIFE-RIFT (*r* = 0.105, *P* = 0.475). But both the groups were comparable on duration of current depressive episode (*P* = 0.705), total number of episodes of depression (*P* = 0.317), and severity of current depressive episode as measured by HDRS-17 score (*P* = 0.191) (Table 2). Global score of LIFE-RIFT had direct significant correlation with HDRS-17 score (*r* = 0.879, *P* = 0.0001).

Mean age of onset of illness was earlier in BAD (24.4 years) as compared to MDD (27 years) and the difference was statistically significant (*P* = 0.026). However, the age of onset in BAD (*r* = −0.058, *P* = 0.694) and MDD (*r* = −0.069, *P* = 0.642) did not show any significant correlation with global LIFE-RIFT score. In both the groups, majority of patients did not have any family history of psychiatric illness and the groups were comparable (*P* = 0.779) (Table 2).

Both the groups were comparable on individual psychosocial domain score but differed significantly in global score (*P* = 0.031) (Table 3). Mean global score for MDD and BAD groups was 15.4 and 16.2, respectively.

In MDD group, most patients showed moderate impairment on psychosocial domain of work (62%), moderate to severe impairment in interpersonal relationship domain (54%), and mild to moderate impairment in domains of life satisfaction and recreation (Table 4). Whereas in BAD group, most of the patients showed moderate to severe impairment in work, interpersonal relationship, and life satisfaction domains and mild to moderate impairment in recreation domain (Table 4).

## 4. Discussion

The main focus of this study was to examine and compare psychosocial functioning in depressed patients of MDD and BAD. It was compared in the domains of work, interpersonal relationship, life satisfaction, and recreation. The results demonstrated an important psychosocial dysfunction among MDD and BAD patients.

Both MDD as well as BAD patients showed impairment in work domain which included employment and household work implying that patients not only neglected household work but also missed their job work leading to absenteeism. Some earlier studies have established the direct relationship between depression severity and job performance [11–14]. In bipolar patients, the correlation between depressive symptomatology and poorer occupational functioning has been reported [15–18]. It has also been found that depressive episodes but not manic or hypomanic episodes were related to poor job functioning [17] and number of depressive symptoms in most recent mood episode predicted unemployment [19].

The difference in work domain was not significant between the two groups. Contrary to our findings, Dean and colleagues reported that rates of employment are low in people with BAD in comparison to those observed in patients with other affective disorders [20].

Earlier work has suggested that functions which depend on traditional roles in family tended to oscillate with the level of depression [21, 22]. In our patients, interpersonal relationships were moderately to severely impaired in both the groups. Nezlek et al. reported that depressed patients, when compared to nondepressed subjects, find their interactions to be less enjoyable and less intimate and feel less influence over their interactions [23].

Both unipolar and bipolar depressed patients had regular arguments with the family members (i.e., spouse or children) and displayed great deficit in emotional closeness. They also avoided seeing the relatives and derived no pleasure in meeting with them. They had no close friends and almost no social contacts. Interpersonal difficulties in depression have been observed by some previous researchers as well [24, 25]. Thus, depressed patients have relationship problems with both close relations and nonintimates and this does not differ by polarity as our results suggest.

Consequently, the patients in both the groups were dissatisfied and showed persistent discontentment with job, family, friends, and finances. They either did not participate in any sort of recreational activities or derived little enjoyment from such activities, if involved in them. Stojanovic-Spehar and Blazekovic-Milakovic while comparing psychosocial factors in depressed and nondepressed individuals found that depressed patients more frequently reported about social isolation, family stress, work stress, and lower life satisfaction than nondepressed patients [26]. A study by Strine and colleagues also reported that there is a strong association between depression, impaired health related quality of life, inadequate social and emotional support, dissatisfaction with life, and disability [27]. Altered quality of life and well-being in depression has also been reported previously [28].

It is clear from our results that all of the BAD patients (i.e., 100%) showed impairment in work, interpersonal relations, and life satisfaction and majority had moderate to severe impairments. Also, they had significantly higher global psychosocial impairment than MDD patients. The previous studies suggest that cognitive impairment is the strongest predictors of psychosocial disability in bipolar disorder [29, 30] and it generates significant disruption to social and vocational adjustment [29, 31–33]. Hence, the cognitive dysfunctions which are proven to be more severe in bipolar depression than unipolar depression [34] are a possible reason for more severe global psychosocial impairment in BAD. Furthermore, goodness of fit between the person and the psychosocial environment is essential for proper psychosocial functioning [35]. Chronic dissonance in this relationship in bipolar disorder has been proposed as a cause of malignant decline in functioning which offers further explanation for our findings in the sense that maladaptive functioning in various psychosocial domains may lead to severe global impairment cumulatively. Here it should be noted that we did not find any significant association between age of illness onset and global LIFE-RIFT score in either of the group, neither was there any significant correlation between number of affective episodes and global LIFE-RIFT score in BAD group. Thus we can assume that age of illness onset and number of affective episode has a little impact on global impairment.

However, the results must be interpreted with caution in view of some limitations of the study. Some of the clinical characteristics like total duration of illness, age onset of illness, and total number of affective episodes were not controlled. The findings cannot be generalized to community as all the patients in our study were recruited from a specialized centre. Though we did rule out the current comorbid axis-1 psychiatric disorders in our patients, but subclinical anxiety symptoms could be a confounding factor. Further studies with larger sample size and longitudinal study design are required to observe how psychosocial functioning varies with the course of each disorder. Also, it would be of interest to analyze the cause and effect relationship between functional impairment and depression.

## 5. Conclusion

In case of depression, although domainwise psychosocial impairment does not differ by polarity, the bipolar depression leads to more significant psychosocial impairment globally. Therefore, the comprehensive assessment of all the spheres of psychosocial functioning, that is, occupation, interpersonal relationships, and life satisfaction, is essential rather than focusing on single isolated domains. This will serve as a guide to propose appropriate psychosocial interventions and achieve complete remission in depressive patients.

## Figures and Tables

**Table 1:** Comparison of sociodemographic profile in MDD and BAD patients.

Variable	MDD (*n* = 48)	BAD (*n* = 48)	*t*/*χ* ^2^ (df)	*P* value
Age (Mean ± SD)	28.1 ± 5.7	27.6 ± 5.5	*t* = 0.46 (94)	0.649
Sex				
Male	37	40	*χ* ^2^ = 0.59 (1)	0.442
Female	11	8		
Marital status				
Married	36	35	*χ* ^2^ = 2.41 (2)	0.389^†^
Unmarried	10	13		
Divorced/separated	2	0		
Occupation				
Unemployed (including house wives)	10	9	*χ* ^2^ = 0.18 (2)	0.914
Professional	19	18		
Farmer/skilled worker/semiskilled worker/unskilled worker	19	21		
Education				
Up to middle	5	5	*χ* ^2^ = 0.19 (2)	0.910^†^
Middle to Sr. secondary	25	23		
Graduate/postgraduate	18	20		
Income				
Nil–6000	20	19	*χ* ^2^ = 1.53 (2)	0.812^†^
6001–15000	25	28		
>15000	3	1		
Religion				
Hindu	46	43	*χ* ^2^ = 1.39 (1)	0.435^†^
Muslim	2	5		
Family Type				
Nuclear	23	26	*χ* ^2^ = 0.39 (2)	0.876^†^
Nuclear extended	23	20		
Others	2	2		
Locality				
Urban	32	28	*χ* ^2^ = 0.71 (1)	0.399
Rural	16	20		

*χ* ^2^: chi-square value; df: degree of freedom; ^†^ *P* value derived from Fisher's exact test.

**Table 2:** Comparison of clinical variables in MDD and BAD patients.

Variable	Mean ± S.D.		*t*/*χ* ^2^ value (df)	*P* value
	MDD (*n* = 48)	BAD (*n* = 48)		
Age of onset of illness (years)	27 ± 5.7	24.4 ± 5.3	*t* = 2.27 (94)	0.026*
Total duration of Illness (months)	15.3 ± 12.6	36.5 ± 24.3	*t* = −5.37 (70.4)	0.000**
Total no. of episodes	1.6 ± 0.8	3.2 ± 1.2	*t* = −7.74 (80.3)	0.000**
No. of episodes of depression	1.6 ± 0.8	1.4 ± 0.7	*t* = 1.01 (94)	0.317
Duration of current depressive Episode (months)	3.85 ± 1.3	3.94 ± 0.9	*t* = −0.38 (83.2)	0.705
HDRS-17 score	17.2 ± 2.3	17.8 ± 2.7	*t* = −1.32 (94)	0.191
Family history				
Present	8	7	*χ* ^2^ = 0.08 (1)	0.779
Absent	40	41		

HDRS: Hamilton Depression Rating Scale-17 item; *χ* ^2^: chi-square value; df: degree of freedom; *P < 0.05; **P < 0.01; ^†^ *P* value derived from Fisher's exact test.

**Table 3:** Comparison of psychosocial functioning in MDD and BAD patients.

Variable	Mean rank		*U* (*Z*)	*P* value
	MDD (*n* = 48)	BAD (*n* = 48)		
(1) Work	44.8	52.2	976 (−1.43)	0.143
(2) Interpersonal relationships	45.4	51.2	1005 (−1.21)	0.255
(3) Life satisfaction	44.8	52.2	972.5 (−1.43)	0.159
(4) Recreation	43.9	53.1	934 (−1.72)	0.085
(5) Global score	42.3	54.5	862 (−2.16)	0.031*

*U* (*Z*): Mann-Whitney *U* value; *P < 0.05.

**Table 4:** Comparison of percentage impaired^a^ in two groups.

Variable	Degree of impairment	MDD (*n* = 48)	BAD (*n* = 48)
(1) Work	Mild Moderate Severe	98^a^ 19^b^ 62^c^ 19^d^	100^a^ 17^b^ 50^c^ 33^d^
(2) Interpersonal relationships	Mild Moderate Severe	100^a^ 8^b^ 54^c^ 38^d^	100^a^ 6^b^ 44^c^ 50^d^
(3) Life satisfaction	Mild Moderate Severe	94^a^ 42^b^ 42^c^ 16^d^	100^a^ 15^b^ 50^c^ 35^d^
(4) Recreation	Mild Moderate Severe	96^a^ 46^b^ 46^c^ 8^d^	96^a^ 28^b^ 54^c^ 18^d^
(5) Global score		100^a^	100^a^

^a^Percentage impaired refers to total number of patients (in %) showing impairment out of total sample of group (i.e., scoring at or above 3 by psychosocial domain and scoring at or above 13 on global score).

^b^Number of patients (in %) showing mild impairment out of total number of patients impaired.

^c^Number of patients (in %) showing moderate impairment out of total number of patients impaired.

^d^Number of patients (in %) showing severe impairment out of total number of patients impaired.

## References

[1] Reddy MS (2010). Depression: the disorder and the burden. *Indian Journal of Psychological Medicine*.

[2] World Health Organization (2004). *The Global Burden of Disease Update*.

[3] Information Bulletins for WorkSafeBC Assessment Providers http://www.worksafebc.com.

[4] Bowlby J (1980). *Loss: Sadness and Depression*.

[5] Tweed DL (1993). Depression-related impairment: estimating concurrent and lingering effects. *Psychological Medicine*.

[6] Judd LL, Akiskal HS, Schettler PJ (2002). The long-term natural history of the weekly symptomatic status of bipolar I disorder. *Archives of General Psychiatry*.

[7] World Health Organization (1992). *The ICD-10 Classification of Mental and Behavioral Disorders*.

[8] Hamilton M (1960). A rating scale for depression. *Journal of Neurology, Neurosurgery, and Psychiatry*.

[9] Leon AC, Solomon DA, Mueller TI, Turvey CL, Endicott J, Keller MB (1999). The Range of Impaired Functioning Tool (LIFE-RIFT): a brief measure of functional impairment. *Psychological Medicine*.

[10] Godard J, Baruch P, Grondin S, Lafleur MF (2012). Psychosocial and neurocognitive functioning in unipolar and bipolar depression: a 12-month prospective study. *Psychiatry Research*.

[11] Adler DA, McLaughlin TJ, Rogers WH, Chang H, Lapitsky L, Lerner D (2006). Job performance deficits due to depression. *American Journal of Psychiatry*.

[12] McKnight PE, Kashdan TB (2009). The importance of functional impairment to mental health outcomes: a case for reassessing our goals in depression treatment research. *Clinical Psychology Review*.

[13] Rytsälä HJ, Melartin TK, Leskelä US, Lestelä-Mielonen PS, Sokero TP, Isometsä ET (2006). Determinants of functional disability and social adjustment in major depressive disorder: a prospective study. *Journal of Nervous and Mental Disease*.

[14] Zimmerman M, McGlinchey JB, Posternak MA, Friedman M, Boerescu D, Attiullah N (2008). Remission in depressed outpatients: more than just symptom resolution?. *Journal of Psychiatric Research*.

[15] Dickerson FB, Boronow JJ, Stallings CR, Origoni AE, Cole S, Yolken RH (2004). Association between cognitive functioning and employment status of persons with bipolar disorder. *Psychiatric Services*.

[16] Fagiolini A, Kupfer DJ, Masalehdan A, Scott JA, Houck PR, Frank E (2005). Functional impairment in the remission phase of bipolar disorder. *Bipolar Disorders*.

[17] Hammen C, Gitlin M, Altshuler L (2000). Predictors of work adjustment in Bipolar I patients: a naturalistic longitudinal follow-up. *Journal of Consulting and Clinical Psychology*.

[18] Kusznir A, Cooke RG, Young LT (2000). The correlates of community functioning in patients with bipolar disorder. *Journal of Affective Disorders*.

[19] Vocisano C, Klein DN, Keefe RSE, Dienst ER, Kincaid MM (1996). Demographics, family history, premorbid functioning, developmental characteristics, and course of patients with deteriorated affective disorder. *American Journal of Psychiatry*.

[20] Dean BB, Gerner D, Gerner RH (2004). A systematic review evaluating health-related quality of life, work impairment, and health-care costs and utilization in bipolar disorder. *Current Medical Research and Opinion*.

[21] De Lisio G, Maremmani I, Perugi G (1986). Impairment of work and leisure in depressed outpatients. A preliminary communication. *Journal of Affective Disorders*.

[22] Wella KB, Stewart A, Hays RD (1989). The functioning and well-being of depressed patients. Results from the medical outcomes study. *Journal of the American Medical Association*.

[23] Nezlek JB, Hampton CP, Shean GD (2000). Clinical depression and day-to-day social interaction in a community sample. *Journal of Abnormal Psychology*.

[24] Altshuler LL, Post RM, Black DO (2006). Subsyndromal depressive symptoms are associated with functional impairment in patients with bipolar disorder: results of a large, multisite study. *Journal of Clinical Psychiatry*.

[25] Marangell LB, Dennehy EB, Miyahara S (2009). The functional impact of subsyndromal depressive symptoms in bipolar disorder: data from STEP-BD. *Journal of Affective Disorders*.

[26] Stojanovic-Spehar S, Blazekovic-Milakovic S (2011). Assessing psychosocial factors in depressed patients—accordance of patient's and physician's assessment. *Collegium Antropologicum*.

[27] Strine TW, Kroenke K, Dhingra S (2009). The associations between depression, health-related quality of life, social support, life satisfaction, and disability in community-dwelling US adults. *Journal of Nervous and Mental Disease*.

[28] Kuehner C, Buerger C (2005). Determinants of subjective quality of life in depressed patients: the role of self-esteem, response styles, and social support. *Journal of Affective Disorders*.

[29] Martinez-Aran A, Vieta E, Torrent C (2007). Functional outcome in bipolar disorder: the role of clinical and cognitive factors. *Bipolar Disorders*.

[30] Bonnín CM, Martínez-Arán A, Torrent C (2010). Clinical and neurocognitive predictors of functional outcome in bipolar euthymic patients: a long-term, follow-up study. *Journal of Affective Disorders*.

[31] Burdick KE, Goldberg JF, Harrow M (2010). Neurocognitive dysfunction and psychosocial outcome in patients with bipolar I disorder at 15 year follow-up. *Acta Psychiatrica Scandinavica*.

[32] Goldberg JF, Burdick KE (2008). *Cognitive Dysfunction in Bipolar Disorder*.

[33] Gilbert AM, Olino TM, Houck P, Fagiolini A, Kupfer DJ, Frank E (2010). Self-reported cognitive problems predict employment trajectory in patients with bipolar I disorder. *Journal of Affective Disorders*.

[34] Murphy FC, Sahakian BJ (2001). Neuropsychology of bipolar disorder. *British Journal of Psychiatry*.

[35] Levy B, Manove E (2012). Functional outcome in bipolar disorder: the big picture. *Depression Research and Treatment*.

